# Associations between major depressive disorder and performance-based and self-reported music cognition

**DOI:** 10.3389/fpsyg.2024.1392710

**Published:** 2024-11-08

**Authors:** Mariana Treviño-Soto, Santiago Gorozpe-Camargo, Álvaro Cejudo-Camarena, María Elena Fernández-Palacios, Ana Claudia Uzárraga-Andrade, Ana Isabel Alamillo-Cuéllar, Aldebarán Toledo-Fernández

**Affiliations:** Facultad de Psicología, Universidad Anáhuac México, Huixquilucan, Mexico

**Keywords:** depression, music cognition, amusia, perception, memory

## Abstract

**Background:**

There is evidence that major depressive disorder (MDD) comes with multiple cognitive impairments including deficits in perception and memory. Music cognition is one of the least explored cognitive functions in relation to MDD, with some studies pointing to mild amusic deficits. These findings, however, are derived only from performance-based tests. Our objectives were to explore differences in music perception and memory between individuals with MDD and a control group, in both performance test and self-report of amusic dysfunction, and to assess the correlation between these measures.

**Method:**

We recruited 62 participants, including MDD individuals (*n* = 34) diagnosed with the Mini International Neuropsychiatric Interview and controls (*n* = 18). All the participants were evaluated with the Montreal Battery for Evaluation of Amusia (MBEA) and the Amusic Dysfunction Inventory (ADI).

**Results:**

None of the assessed dimensions from the MBEA or the ADI showed statistical differences between groups. Some significant associations were found between ADI's Vocal Production and the MBEA's three tests of the melodic dimension (Scale, Contour and Interval) and between MBEA's Scale and Memory, Meter and ADI's Melodic Perception, and tests of Memory from each respective instrument.

**Conclusion:**

Results suggest that perception and memory of basic music stimuli are not among the cognitive deficits within MDD, however, they may be indirectly affected by other cognitive phenomena common to this psychopathology, such as poor sustained concentration due to mental fatigue.

## Introduction

Major depressive disorder (MDD) is commonly understood as an affective psychopathology characterized by feelings of deep sadness, anhedonia and apathy which may impair a person's functionality within its everyday-life activities (American Psychiatric Association, 2013). A relatively more recent literature on MDD, also understands it as a disorder with multiple cognitive manifestations besides deficits in concentration which is one of its secondary diagnostic criteria. Indeed, MDD has been directly associated with mild-to-moderate deficits in working memory, executive function, processing speed and episodic memory (Millan et al., 2012; Rock et al., 2014), and with corresponding brain anomalies such as decreased cortical thickness in orbitofrontal and temporal cortices (Li et al., 2020), global hippocampal atrophy (Santos et al., 2018), and overall altered dynamics of brain networks (Liu et al., 2022).

One of the least explored cognitive functions in relation with MDD is *music cognition*. This refers broadly to a person's abilities to perceive, encode, retain, and manipulate music stimuli (sounds organized in time that do not depend on verbal content or visuospatial information). Arguably the most recognized model of music cognition is the one proposed by Peretz et al. (2003), dividing it into a *melodic dimension* involving perception of contour, interval and scale, and a *temporal dimension*, involving perception of rhythm and meter. For both dimensions, a *memory* component in the model accounts for the capacity to recognize melodies previously stored as a music repertoire. The term *amusia* is used to describe deficits in perception or memory of any of the musical dimensions (though most often the melodic one) and its etiology has been proposed to be congenital or acquired via brain lesion (Sihvonen et al., 2019; Szyfter and Wigowska-Sowińska, 2022), both being relatively low in their prevalences (Pfeifer and Hamann, 2015; Peretz and Vuvan, 2017).

The relatively low interest in the association between music cognition and MDD may be because researchers perceive music cognition as less crucial for daily-life functioning of the person with MDD, being other cognitive processes such as attention and memory far more relevant, especially with non-musicians; as well, being MDD a mood disorder, most of the studies have centered around the relationship between music and emotions, and not cognition. From this branch of research, are particularly informative the ones evaluating the effect of music therapy in individuals with MDD. A recent meta-analysis (Tang et al., 2020) reviewing 55 randomized clinical trials, found overall strong effects of music therapy on depression, dependent on the therapy methods, with recreative music therapy and guided imagery showing superior effects on the reduction of symptomatology. These findings highlight the strong connection between music and the regulation of mood; however, they do not share light on the functioning of basic cognitive processes in individuals with MDD. Indeed, most of the interest in the neurobiology of music cognition, especially among individuals with MDD, have been directed toward emotional functioning, particularly the effect that music has on emotions, and not the other way around. Neuroimaging studies have found that music stimuli directly engage limbic and paralimbic structures; for example, brain areas involved in reward processing such as the nucleus accumbens, and structures involved in the generation and modulation of negative emotions (amygdala) and positive emotions (hippocampus) (Koelsch et al., 2010).

Evidence for alterations of music cognition in individuals with MDD have been reported, including tonal (Tollkötter et al., 2006) and emotional processing (Osuch et al., 2009) just to name a couple. A relatively recent study (Reker et al., 2014) reported significant deficits of musical ability in patients with MDD, with some of these individuals (28.6%, *n* = 21) even qualifying for diagnosis of amusia. Of particular interest from this study was the fact that musical abilities seemed to improve after remission of depressive symptoms at around one-month follow-up, highlighting the possible causal association between MDD and deficits in music cognition. In general, these authors attribute the deficits in non-emotional tests of music perception as byproducts of attentional and working memory impairments.

Another recent study (Raghavendra et al., 2022) following the hypothesis that impaired attentional functions may have detrimental effects upon other processes such as perception and memory, found no significant differences between healthy controls (*n* = 18) and MDD individuals (*n* = 19) regarding music perception and memory. As an alternative explanation to these findings, the authors suggest that the recruited MDD group proved preserved global cognitive abilities, which may have prevented the observation of the detrimental effects of this disorder upon music cognition.

One major caveat from the studies exploring the association between MDD and impairment of music cognition is the total reliance on performance-based tests. These types of measurements are a definitory part of the neuropsychological assessment since they provide objective information from sampling simplified cognitive behavior; however, given their often-limited ecological validity, other sources of information, such as self-reports, should be addressed when evaluating a neuropsychological condition, to better integrate the diagnosis by obtaining subjective information about everyday-life experiences of the individuals. Indeed, evidence of associations between performance-based tests and self-reports of different components of music cognition have been reported (Toledo-Fernández et al., 2018), though not in the context of MDD.

The current study aimed at exploring the association between MDD and music perception and memory, using both an objective performance test and a subjective self-report of amusic deficits. We hypothesized that, compared to a non-clinical control group, (1) individuals with MDD will prove lower music perception and memory scores in a performance-based test, (2) will self-report higher everyday amusic dysfunctions; and (3) that both sources of measurement will show theoretically expected correlations across both groups (e.g., performance on task of pitch perception will negatively correlate with self-reported difficulties in everyday-life melodic perception).

Overall, we believe that the findings of the study will provide further evidence on the relations between MDD and music cognition, advancing the understanding on the cognitive impairments characterizing this psychopathology.

## Method

### Participants

The sample was recruited non-probabilistically via the distribution of different flyers in social media describing the basic eligibility criteria needed to be enrolled in the study. The first recruitment process was aimed toward potential cases of MDD, starting in August and ending in late October 2022. The eligibility criteria for the cases to begin the comprehensive psychiatric interview were having experienced feelings of sadness or loss of interest in daily activities most of the time within the last 2 weeks, as well as the requirements of not having any neurological or psychiatric disorder in the past 12 months, and not having diagnosis or suspicions of hypoacusia. Furthermore, for the control group, flyers were used to recruit participants who reported not having experienced feelings of sadness or loss of interest in daily activities most of the time within the last 2 weeks, that did not have a diagnosis of any mental disease nor had any hearing problems. The demographic characteristics of the control group were intended to kept paired with regards to age and gender by directing the invitation flier within respective social groups. This recruitment was conducted parallel to the recruitment of the cases.

### Instruments

#### Montreal battery of evaluation of amusia

This battery was used to evaluate music perception and memory, as it is the gold standard for evaluation of deficits of music basic cognition. The MBEA is composed of six subtests that assess six music processing components: Scale, Contour, Interval, Rhythm, Metric, and Music Memory. All six tests use the same pool of 30 novel musical phrases. The first four subtests (Scale, Contour, Interval, Rhythm) start with two practice trials and 30 experimental trials, each trial is preceded by a warning tone, a target melody, then a comparison melody, that are separated by a 2 s silence. In addition to those 30 trials, each subtest contains a catch trial to ensure that the participants are paying attention. In all these subtests, subjects are required to judge on each trial whether the target and the comparison melody are the same or not. For the fifth subtest, which is preceded by four practice trials, the participants had to judge whether the presented melody is a march or a waltz. Finally, the Memory subtest presents only single melodies, half of which already occurred in the previous subtests, the other half is new, and participants had to indicate for each melody whether they have heard it before during the previous subtests or not (Peretz et al., 2003).

The MBEA provides sum scores corresponding to each of the six subtests and a global score obtained by the mean of these results. Higher scores are interpreted as normal functioning of music cognition. As described by Peretz et al. (2003) individuals' performance on the MBEA tends to show a ceiling effect, with most of them obtaining scores above 25 of the 30 points that can be obtained.

Evidence on the validity of the MBEA across several cross-cultural samples have accumulated since its first publication (Pfeifer and Hamann, 2015; Peretz and Vuvan, 2017; Paraskevopoulos et al., 2010), including Mexican samples (Toledo-Fernández et al., 2018; Toledo-Fernández and Salvador-Cruz, 2015).

#### Mini international neuropsychiatric interview

The MINI is a structured interview for diagnosing psychiatric disorders according with the DSM-IV criteria (Sheehan et al., 1998), and it is designed to allow for administration by non-specialized interviewers in a relatively short time. For this study, a Spanish-translated version was used to diagnose current MDD episode only. The first two items of this interview are meant to detect if the respondent endorses at least one of the primary indicators of MDD (feelings of sadness and loss of motivation in the last 2 weeks); when not endorsing any, the interview is stopped and thus the secondary criteria (e.g., changes in eating of sleep habits, etc.) are not explored.

Although based on a past version of the DSM, the diagnostic procedure for MDD within the MINI is still valid, and the interview has been used in several studies in Mexican population (Marín-Navarrete et al., 2018; Pérez-López et al., 2018).

#### Amusic dysfunction inventory

The ADI is based on the MBEA's dimensions of music perception and memory, and on complaints of daily life impairments that are often reported by individuals with amusia. The ADI consists of nine items divided in four domains that aim to evaluate complaints of daily life amusic impairments according to four dimensions: Melodic Perception, Rhythmic Coordination, Vocal Production, and Memory. It is structured in a Likert-type scale from “Never” = 1 to “Always” = 4. All items and answer possibilities have been translated from Spanish (Toledo-Fernández et al., 2018). Sum scores for each of the domains are computed with higher scores indicating more amusic difficulties (Table 1).

**Table 1:** Items of the amusic dysfuction inventory.

**Melodic perception**
1. I can tell when an instrument is out of tune.
2. I am capable of clearly noticing an incorrect note in a familiar melody.
**Rhythmic coordination**
3. I dance fluently and to the rhythm of the music.
4. It is difficult for me to follow the rhythm of a song with my hands or my feet.
**Vocal production**
5. People say I sing out of tune.
6. I sing out of tune.
**Memory**
7. I have difficulty in recognizing the melody of a song when there are no lyrics.
8. I have trouble remembering melodies that I have heard several times before.
9. I can only remember the lyrics of the songs and I often forget the melodies.

Criterion validity of this inventory was explored in its original study, proving associations between MBEA's subtests Scale, Meter and Memory and self-reported capacity for Melodic Perception, Vocal Production, Rhythmic Coordination and Memory (Toledo-Fernández et al., 2018).

### Procedure

For the recruitment of the samples, two different flyers were designed describing the basic eligibility criteria for both the cases and the control group, and the contact information of the research team. The distribution was done through social media and paid publicity on Facebook to reach all the Mexican territory. Once a candidate was interested and had contacted the research team, a date was accorded for the evaluation.

During the session, which lasted around 1 h and 40 min, the interviewer applied all the measures starting with a demographic questionnaire, followed by the MINI, the MBEA and the ADI. At the end of the evaluation, the interviewer returned the results to the participant if he/she agreed and asked him/her to share the flier through social media platforms.

The study protocol was approved by the Ethics Committee of the Universidad Anáhuac México. All participants signed informed consent after careful reading with the interviewers before collecting any data, and all the evaluations were performed in the presence of the lead researcher (PhD in Clinical Neuropsychology), whom also provided rigorous training to the interviewers, including several modellings and rehearsals of the protocol before beginning data collection.

### Statistical analysis

Descriptive statistics included mean and standard deviation for numerical variables, and frequencies and percentages for categorical ones. Chi-square test and Mann-Whitnney's *U* were utilized to compare control and case groups for variables with respective levels of measurement. Results of the MBEA and the ADI were correlated using Spearman rank test.

To determine statistical significance, *p* < 0.05 was set as the criterion. All the analyses were performed using JASP version 0.16.

## Results

Table 2 displays the characteristics of the samples, showing that both groups were mostly constituted by young adult (ages between 18 and 53 years old) women, both groups by high school-to-graduate educational attainments, and almost half of the sample with no music training. There were no significant differences between groups within these variables and, indeed, only age differed between groups, being the control the eldest one. Regarding music cognition, none of the assessed dimensions from the MBEA or the ADI showed statistical differences. It can be noticed that the Interval test showed the lowest score in individuals with MDD relative to the control group; as well, the Rhythmic Coordination, Vocal Production, and Memory dimensions of the ADI showed higher scores, suggesting a tendency toward amusic difficulties.

**Table 2:** Descriptive statistics and differences between groups.

	**Cases (current MDD) (*N* = 34)**	**Control (no MDD) (*N* = 28)**	***p-*value for difference tests^b^**
	**Mean (SD) or** ***n*** **(%)**	**Mean (SD) or** ***n*** **(%)**	
Gender^a^			0.74
Male	10 (29.41)	10 (35.71)	
Female	22 (64.70)	18 (64.28)	
Age	25.76 (6.81)	31.78 (11.03)	0.03
Educational attainment			0.08
High school	17 (50.00)	7 (25.00)	
Graduate	15 (44.11)	16 (57.14)	
Post-graduate	2 (5.88)	5 (17.85)	
Music training			0.37
None	19 (55.82)	14 (50.00)	
1- to −2 years	9 (26.47)	5 (17.85)	
>2 years	6 (17.64)	9 (32.14)	
**MBEA**			
Scale	24.76 (3.34)	25.35 (2.92)	0.62
Contour	25.08 (3.03)	24.57 (3.50)	0.53
Interval	22.88 (2.83)	24.00 (3.27)	0.15
Rhythm	25.94 (2.65)	26.28 (2.52)	0.71
Meter	25.29 (4.77)	25.82 (4.01)	0.75
Memory	27.14 (1.97)	26.82 (2.40)	0.63
Global	25.18 (1.95)	25.47 (2.15)	0.83
**ADI**			
Melodic perception	4.50 (1.39)	4.50 (1.47)	0.86
Rhythm	3.88 (1.51)	1.03 (1.75)	0.84
Vocal production	5.47 (1.72)	4.85 (1.45)	0.16
Memory	5.41 (1.28)	5.00 (1.49)	0.16

^a^Two participants preferred not to respond.

^b^Chi-square test for categorical variables, and Mann Whitney's *U* for numerical variables.

ADI, Amusic Dysfunction Inventory; MBEA, Montreal Battery of Evaluation of Amusia; MDD, major depressive disorder.

As it can be seen in Table 3, significant associations were found between the ADI dimensions and the MBEA tests, mainly between Vocal Production and the three tests of the melodic dimension (Scale, Contour and Interval) and between Scale and Memory, Meter and Melodic Perception, and tests of Memory from each respective instrument.

**Table 3:** Spearman rank correlations between results from the MBEA and the ADI (*N* = 62).

	**Scale**	**Contour**	**Interval**	**Rhythm**	**Meter**	**Memory**	**Global**
Melodic perception	−0.23	−0.03	−0.14	0.14	−0.33^**^	0.06	−0.20
Rhythmic coordination	0.03	0.26^*^	0.18	−0.03	−0.01	−0.01	0.14
Vocal production	−0.29^*^	−0.33^**^	−0.34^**^	−0.03	−0.14	−0.10	−0.35^**^
Memory	−0.35^**^	−0.10	−0.22	−0.17	−0.16	−0.29^*^	−0.29^*^

^*^p < 0.05; ^**^p < 0.01.

ADI, Amusic Dysfunction Inventory; MBEA, Montreal Battery of Evaluation of Amusia.

## Discussion

The main objective of the study was to explore deficits in music perception and memory in individuals with MDD through performance test and self-report. No significant difference was found between these individuals and the control group with no MDD. Even though it is known that MDD tends to interfere with certain cognitive abilities including perception and memory of different modalities (Millan et al., 2012; Rock et al., 2014), it is possible that, due to the MBEA's ceiling effect (Peretz et al., 2003), we couldn't detect subtle music deficits in the individuals with MDD of our sample. This finding is similar to the one reported by Raghavendra et al. (2022) who also found no difference between control and cases on the MBEA, explaining it by the lack of general cognitive impairment of their cases according to assessment with neuropsychological performance tests.

It may well be that the use of maximum performance tests, which tend to display normal distribution of performance across large samples, could reveal subtle deficits in music perception and memory. For example, Reker et al. (2014) did find poorer music abilities in individuals with MDD using a set of tests that, according to their description, appear to be slightly more difficult than the subtests of the MBEA; e.g., the melodic comparison test, which is similar to the first three subtests of the MBEA, included variations of more than just one note, whereas the MBEA only uses one variation per stimuli. Also, in the Rhythmic test used by these authors, the participant is asked to reproduce by tapping or clapping a rhythmic pattern, which could indeed be a more difficult task than just recognizing.

Within the MBEA, the only test that showed slightly higher tendency toward a lower score was the Interval test, which evaluates the perception of the distance between two musical notes. Because this test is the third one in sequence within the MBEA, and since by this point the responder has gone through two almost identical tests and around 20 min of evaluation, we hypothesize that this slight tendency might be explained by cognitive fatigue, which refers to a decrease in execution during a cognitive task that requires sustained mental effort. Vulnerability toward fatigue, including mental one, is a common staple of depression (American Psychiatric Association, 2013), so we believe that our participants with MDD may have experienced this fatigue because of the repetitiveness and duration of the tests. Our supposition is strengthened by the fact that the test that follows Interval, the Rhythm test, implies an important variation of the stimulus, which may regain the attention of the respondents.

Finally, with regards to the first objective of the study, differences between groups were neither found in the four dimensions of the ADI. These results may serve as supporting evidence for the retention of our null hypothesis, meaning that MDD individuals do not show impairment in music perception and memory. However, considering our previous hypothesis explaining the normal performance of MDD individuals in the MBEA, it could also be that the music perception and memory deficits may be so subtle that they are not even consciously registered by the individual.

The second objective of our study was to test the association between the performance-based test, the MBEA, and the self-report of amusic complaints, the ADI. This objective was considered aiming to strengthen the findings on the music cognition of the MDD individuals, expecting a negative correlation between these two instruments within the total sample (because, if the tests are indeed valid, the covariations between them must be independent of the samples). In fact, we did find these correlations, although some of them with an irregular pattern. On the one hand, ADI's Vocal Production mildly correlated with the three melodic tests of the MBEA, which could be explained because the correct intonation when singing requires the constant adjustment of the vocal tract based on the feedback from the perception of one's voice and the accompanying music. As well, the Memory dimension from both tests also correlated mildly. The observed strength of the correlation may be explained by the limited ecological validity found in some neuropsychological tests (Chaytor and Schmitter-Edgecombe, 2003); this is, there is an important behavioral distance between the performance of an individual within an artificial test and his/her performance in real-life scenarios.

Other associations between the MBEA and the ADI are less clear. Although speculative, we believe that they could be based on the perception of the respondents of their own performance within these MBEA's Scale and Meter (Table 3), which are salient in two ways: the Scale test is the very first one of the MBEA, and the Meter test is the one with a different task relative to the other subtests. In this way, respondents may remember more clearly their performance in these subtests, especially when self-perceived as a poor one, and subsequently judge more negatively their own everyday-life music difficulties, as measured with the ADI. This, of course, is a limitation within our study, since we did not randomize or alternate in any way the sequence of the instruments administered to the participants.

## Limitations

The first limitation that we can identify in our study is the use of a performance test for music cognition with a high ceiling effect on its scores (Peretz et al., 2003), the MBEA, which can be suitable for the sensitive identification of clear cases of amusia and not for the profiling of subtle music perception and memory deficits. We used the MBEA as it is currently the gold standard for assessment of impairments in music cognition and because of its availability as an open-access measurement. Also, we intended to back up the evaluation of our participants using the self-report ADI.

The second most important limitation was the sample size, which could have prevented the identification of significant differences between cases and the control group; this is of particular relevance considering that the effect size in the association between MDD and music perception and memory may be rather mild, since MDD does not suppose a clear and specific brain lesion within cortical structures related to music cognition. However, this sample size is not uncommon in studies of this topic (Raghavendra et al., 2022; Reker et al., 2014) and we tried to ensure the selection of real MDD cases with the use of a valid and reliable psychiatric measure, the MINI. On the other hand, considering that one of MDD's main symptoms is lack of interest in everyday life activities (American Psychiatric Association, 2013), it is uncertain why individuals with this pathology were interested in participating in the study when recruited via social media. This leads us to believe that perhaps cases presented a less severe MDD (even when detected by the MINI), and possibly explain why there were no differences with the control group regarding music perception and memory.

Also, in relation to the sample, although matching of cases was intended for age and gender, if was not possible to control optimally, thus having significant between-group differences with regards to these demographic variables. However, most of the participants fall under the category of young adults which prevents that any significant difference in basic cognition (such as music perception and memory) could be observed. Concerning gender, although more female participants were recruited, no significant differences between genders have been reported in the literature with the MBEA as to suspect that the data was significantly affected by this demographic and, besides, it is indeed the most common gender participating in this line of research (PFEIFFER).

Finally, the last relevant limitation had to do with the logistic restrictions imposed by the COVID-19 lockdown, which was going on by the time we started the participants' recruitment. Consequently, the instruments had to be administered via online video services like Zoom, which implies the risk of diminishing the participants' performance due to potential audio and internet connectivity issues. Considering this, we made sure to have a stable internet connection throughout the whole test administration, especially for the MBEA, and asked the participants to procure the same, not having any report of sound issues during the administrations.

## Conclusions

To our knowledge, this is the first study addressing the relationship between MDD and basic music cognition in a Mexican sample, and it is one of the few recently conducted studies found in the literature. We believe that our findings add to the general knowledge about cognitive deficits within MDD, suggesting that perception and memory of basic music stimuli is not among them, but may be indirectly affected by other cognitive phenomena common to MDD, such as mental fatigue or lack of concentration, though with a weak effect size. Further studies should explore this association with the use of more sensitive tests of music cognition deficits.

## Data Availability

The raw data supporting the conclusions of this article will be made available by the authors, without undue reservation.
